# Implantation, with Clinical Demonstration

**Published:** 1888-06

**Authors:** M. H. Fletcher


AMERICAN JOURNAL

—OF—

DENTAL SCIENCE.

VOL. XXII. THIRD SERIES—JUNE, 1888. NO. 2.

ARTICLE I.

### IMPLANTATION, WITH CLINICAL DEMONSTRATION.

[Read before the meeting of the Mississippi Valley Dental Association, held at Cincinnati, Ohio, March 8, 1888, by M. H. Fletcher, D. D. S., M. D.

The operation of implantation, as recently advocated and brought into use by Dr. Younger, of California, is not more interesting from its practical value—whatever that may be—than from the physiological processes which are exited into action during the repair or healing of a successful case.

Dr. Younger claims that implanted teeth are excepted and adopted by the adjoining tissues in proportion to the amount of their surfaces covered by peridental membrane. He thinks that in the absence of this membrane the implanted tooth is always rejected and the operation in such cases is always a failure. The peridental membrane, the Doctor claims, is revivified by being surrounded again with its natural environment. He believes that the membrane will thus revive even after the laspe of years, provided there has not been such exposure as would change its organic character or cause disintegration. He alludes to the well-known fact that various forms of spores and bacteria are known to lie inactive for indefinite periods of time, and then, when brought again into the proper environment, to revive, grow and proliferate. He claims that the reviving of the peridental membrane on implanted teeth is of a character analogous to this and that to this essential fact is due the success of such operations.

In opposition to this view of Dr. Younger and his supporters, many others (the writer having been one of them) have claimed that there is no union of tissue, but that implanted teeth are held in position simply by that form of articulation known as "Gomphosis," just as a nail is held in place when driven into wood. When the implanted tooth is crowded into position there is sufficient contact between the walls of the prepared socket and the roots of the tooth to hold it in place temporarily. It is of course impossible to prepare a socket which will exactly fit the irregularities of the root of a tooth. But after the tooth is in place, cicatricial tissue begins to grow and fills up the spaces intervening between the root of the tooth and the surrounding wall of bone. In this way the force or pressure, described as gomphosis, is brought to bear evenly over all parts of the root and the tooth is held firmly in position. It is undoubtedly true that normal teeth are retained in place in the same way, but whether gomphosis constitutes the entire fastening or not is a mooted question. The writer formerly believed it was, and at a meeting of the Cincinnati Odontological Society held last June, advocated the covering of the roots of implanted teeth with lead, pure gold, or some other such substance, which could not be absorbed by the circulation, believing that were this not done, absorption of the root would be the ultimate result in the majority of these operations; as it is in cases of transplanting and replanting. A dentist of California

(whose name did not appear) is reported in one of our late journals as advocating this theory of covering with lead as being the best method of preserving the root from destruction. He has inserted some teeth thus covered, but with what success I have not learned.

Having had the pleasure of meeting Dr. Younger during the past summer, the writer had quite a lengthy talk with him concerning this topic. The doctor, being of a good-natured temperament, bore patiently with the persistence of your humble servant while the latter was arguing earnestly in favor of his theories against the Doctor's practical experience.

My own experience in implanting is quite limited, including but three cases all told. But in this limited experience there has been sufficient evidence that my former theories were incorrect. In one of these cases, the operation having proved a failure to a great degree, the tooth was extracted after having been in position over four months. Upon examination it was plainly to be seen that a considerable attachment had already been formed on one side by the peridental membrane. This tooth (a superior lateral) was implanted the 4th of last October, the original tooth having been lost about ten years previously from pyorrhœa alveolaris. The space between the canine and the central had closed to some degree so that it was necessary to select a very small lateral to insert between them. The only tooth available small enough for the place had been partially denuded of periosteum from calcareous deposits as well as by scraping. At that time, however, I did not deem this a matter of consequence. The tooth having been implanted it was cemented to the adjoining teeth by oxyphosphate. It progressed very nicely for about three weeks when pus began to exude from under the gums on the labial surface. This continued in varying quantities up to the time of extraction, in spite of all local treatment. It was a matter of much surprise to me, and especially under the circumstances of so free a discharge of pus, to find that a considerable zone of peridental membrane had attached itself to the bone. This attachment was so marked as to cause considerable pain in extracting.

These facts of attachment and pain on extracting are identical with those observed by Dr. Younger himself in his own personal experience, he having had an implanted tooth in his own mouth for the space of seven months, and then having had it extracted for the purpose of having the membrane examined. He experienced considerable pain in the removal, in consequence of its having been quite firmly attached.

The tooth of which you have had the history in connection with my own practice is here for your examination. The portion covered with membrane is easily distinguished from the other surface.

Now, while these two or three teeth which have been extracted and were found to have become attached to the bone do not prove conclusively Dr. Younger's theory of the reviving of the perio-dental membrane to be true in every case, yet these, with the numerous other cases of his and others, which have been implanted and have become attached and seem to be entirely successful, indicate very strongly that such is the normal tendency and result in all cases properly operated upon, and this form of repair of tissues is becoming an accepted fact in physiology.

It must be remembered that in this process of repair, after implantation, that time is necessary for the work to be done As to the length of the time of repair and the continuance of the accompanying symptoms we have only to recall the clinical history of the recovery of a fractured bone in other parts of the body; this we know varies in different persons and is controlled by circumstances and conditions. It should be borne in mind also that such cases are often attended with more or less traumatic fever, and the appearance of that symptom in connection with the operation of implantation should not occasion surprise.

I have two other cases to present, both of which are doing well, one having been implanted last May and the other the fifteenth of November last. The May case is a right superior bicuspid. The original tooth having been extracted only three months, there was a small depression where the socket had been and the absorption of the alveolus had not been very great. But the new socket had to be drilled into the bone much the same as though the tooth had been out for years. The new tooth was kept cemented in position most of the time for about four weeks and has become perfectly firm, as you may see by examination.

The November case is also a superior bicuspid on the right side. This was left without ligature, cement, or any kind of fastening, in order to determine, if possible, whether fastening was necessary. It has not done so well as the preceding case, but still is doing well enough to remain and to be useful. At the time of this operation I took from the place where this tooth now is, about the eighth of an inch of the apex of the old bicuspid which was fastened only to the gums.

It might be said by some that these cases were simply transplanting, but they are far from it, for in each one a new socket was made for the reception of the implanted tooth.

In reviewing the history of the few operations of this kind, we conclude as to the point of practically, that we may certainly class implantation as among the legitimate operations coming under the head of Dental Surgery.

Many persons have mistakenly supposed the operation of implanting is very similar to transplanting and replanting. The fact is that it differs from both these operations in almost every particular. To discuss this matter fully would require a special paper. But a point or two in this connection may be suggested here. About the only similarity in the three operations is the fact that a natural tooth, the root of which is still covered with periodental membrane, is used in each. In transplanting and replanting, a tooth is fitted into the natural socket of the original tooth

But in implanting, a new socket is cut into the maxillary bone by drilling into its substance. This operation opens the haversian canals in the dense portion of the bone and also penetrates into the medullary spaces in the cancellous portions. Since it is not practicable to prepare a socket that will fit exactly the depressions and irregularities on the root of the tooth to be inserted there must necessarily be more or less space left between the root and the surrounding tissues, after the tooth is placed in position. This space, or we might say, these spaces, are at once filled with blood from the freshly opened blood-vessels in the bone. The usual granulations begin to form and as they approach the surface of the tooth they come into contact with an old friend as it were in the periodental membrane with which it is covered. This membrane, under what is so nearly its original natural environment of blood supply, etc., is supposed now to revive and enter upon new and continued life. The process of granulation proceeds until all the vacant space in the new socket is filled up. The old membrane on the tooth furnishes the cells which are supposed to proliferate, and what may be termed a new periodental membrane is thus formed. If this theory of the method of the acceptance and retention of the new tooth is correct, it is obvious that the operation of implanting is much more likely to be successful than either that of transplanting or replanting. In both the latter operations the walls of the original socket are left intact. These are penetrated by but few blood-vessels, and these are confined principally to one portion of the socket, viz.: the apex. There is therefore but slight opportunity for granulation. The blood which flows into the vacant spaces in the socket deteriorates. The periodental membrane and fragments of tissue on the transplanted or replanted tooth under these conditions must degenerate. The consequence is that pus is formed, and the operation is most likely to be a failure. But the conditions are different when the walls of the old socket are cut away. Numerous blood-vessels are thus opened in every part of the socket, and the process of granulation begins about every part of the root of the new tooth. Nature in thus repairing damages builds a new socket for the tooth and the operation becomes successful.—*Dental Register.*

---

ARTICLE II.

### A CASE IN OFFICE PRACTICE.

